# Efficacy of perioperative high-dose prednisolone therapy during thymectomy in myasthenia gravis patients

**DOI:** 10.1186/1749-8090-8-226

**Published:** 2013-12-10

**Authors:** Yoshito Yamada, Shigetoshi Yoshida, Hidemi Suzuki, Tetsuzo Tagawa, Takekazu Iwata, Teruaki Mizobuchi, Naoki Kawaguchi, Ichiro Yoshino

**Affiliations:** 1 Department of General Thoracic Surgery, Chiba University Graduate School of Medicine, 1-8-1 Inohana, Chuo-ku, Chiba 260-8670, Japan; 2 Department of Neurology, Chiba University Graduate School of Medicine, Chiba 260-8670, Japan

**Keywords:** Myasthenia gravis, MG crisis, Prednisolone, Thymectomy

## Abstract

**Background:**

This study aimed to investigate the benefits of administering perioperative high-dose prednisolone in conjunction with thymectomy in patients with myasthenia gravis.

**Methods:**

We retrospectively reviewed data from patients with Myasthenia Gravis Foundation of America Clinical Class I to IIIB who had undergone an extended thymectomy between 1992 and 2009. Perioperative high-dose prednisolone was administered at starting doses of 10 to 20 mg and escalated up to 100 mg on alternate days. The treatment group comprised 70 patients receiving perioperative high-dose prednisolone, whereas the control group included 61 patients not treated with preoperative steroids. The two groups were compared with respect to baseline clinical characteristics, incidence of postoperative complications, and follow-up disease status.

**Results:**

Prednisolone-treated patients presented with more advanced disease compared to controls (Class IIB or greater, 42 [60.0%] versus 7 [11.3%], respectively; *P* < 0.001). Mean preoperative%FVC was lower and FEV1.0% was higher in treated patients than in controls (%FVC: 92.4 ± 2.3% versus 99.5 ± 2.4%, respectively; *P* = 0.037, FEV1.0%: 85.2 ± 1.3% versus 81.4 ± 0.9%, respectively; *P* = 0.017). The groups were similar in other variables including presence of thymoma, and operative procedure. In the treatment group, disease status was significantly improved only by the induction of high-dose prednisolone before the surgery (*P* < 0.001), and these patients discontinued anti-cholinesterase therapy more frequently than controls (*P* < 0.001). Moreover, the treatment group demonstrated markedly lower rates of postoperative crisis (12.2% versus 2.9%, respectively; *P* = 0.045). The incidence of infection, wound dehiscence, and diabetes mellitus were comparable between groups. Survival analysis demonstrated higher rates of treated patients with improved disease status at three and five years (92% and 96%, respectively) compared to controls (57% and 76%, respectively; *P* < 0.001). Likewise, significantly greater proportions of treated patients achieved complete stable remission or pharmacologic remission at three, five, and ten years (23%, 42%, and 72%, respectively) compared to controls (10%, 20%, and 44%, respectively; *P* = 0.002).

**Conclusions:**

Perioperative high-dose prednisolone therapy is a safe, promising strategy for managing patients with myasthenia gravis and may reduce the incidence of postoperative crisis while improving disease status.

## Background

Myasthenia gravis (MG) is an autoimmune disease caused by acetylcholine receptor antibodies (AchR-Ab) that block acetylcholine receptors (AchR) at the postsynaptic neuromuscular junction. In 1935, Simon demonstrated the efficacy of percutaneous adrenocorticotropic hormone [1] and in 1939 Blalock and colleagues espoused the value of thymectomy [2]. Subsequently, further advancements in immunosuppressive therapy and thymectomy have formed the mainstay of treatment for MG [3,4]. However, given the paucity of strong evidence supporting the utility of thymectomy in the management of MG, standard treatment guidelines have not yet been established [5].

In our institution, preoperative stabilization of MG is considered essential for preventing postoperative disease crisis and achieving early remission. Thus, a multimodal approach including a regimen of prednisolone (PSL) combined with extended thymectomy has been proposed. In 1978, we implemented a strategy of preoperative high-dose PSL therapy followed by extended thymectomy for MG patients, which produced favorable results [4,6]. However, whether administering a perioperative regimen of steroids during thymectomy confers superior benefits over thymectomy alone remains undetermined. Therefore, we conducted a retrospective analysis in order to investigate the clinical benefits of perioperative high-dose PSL administered as an adjunct to extended thymectomy.

## Methods

### Patients

We retrospectively reviewed records from 180 patients who had been diagnosed with MG and had undergone extended thymectomy between 1992 and 2009. Detailed data were prospectively collected at the time of hospital discharge and from outpatient charts, and included information on patient characteristics, medical history, neurologic findings, disease status, the presence of thymoma, use of immunosuppressive therapies (i.e., PSL or tacrolimus) and anti-cholinesterase agents, induction with plasma exchange, operative procedures, postoperative complications, pathologic diagnosis, and follow-up disease status. Regarding the patients evaluated before the year 2000, when the Myasthenia Gravis Foundation of America (MGFA) classification was introduced, we redefined the MGFA class according to the clinical records, including thorough data of neurologic findings. We included patients considered to have MGFA Class I to IIIB and excluded very severe cases, such as MGFA Class IV or V, because the variability and complexity of therapies for these latter cases made it difficult to evaluate the efficacy of the perioperative prednisolone therapy alone. Eligible patients were divided into two groups. The ph-PSL group comprised patients who had received perioperative high-dose PSL according to the protocol described below. The control group consisted of patients who had not received steroidal treatment preoperatively. Patients with a history of immunosuppressive agent use such as steroids were also excluded; only patients who were administered steroids as part of the “preoperative high-dose prednisolone therapy protocol” were included in the research group. The two groups were compared on the basis of clinical characteristics, the incidence of postoperative MG crisis and other complications, and follow-up disease status.

### Indication for treatment with high-dose PSL

After the diagnosis of MG, informed consent was obtained from each patient and medical treatment was initiated. Depending on the patient’s age, clinical history, MG status, and the presence of thymoma, the neurologists considered therapeutic options including the administration of anti-cholinesterase, high-dose PSL, thymectomy, intravenous immunoglobulin, and/or plasma exchange. Patients with less severe MG such as MGFA I or IIA were likely to undergo only thymectomy with or without anti-cholinesterase. In cases with advanced thymoma, thymectomy was considered a priority, and high-dose PSL was not administered. Patients with severe MG status, including MGFA IIB or more were likely to have ph-PSL therapy after obtaining the patients’ consent.

The operative method was extended thymectomy in all cases, regardless of open chest surgery with sternotomy or video-assisted thoracoscopic surgery (VATS). Extended thymectomy consisted of thymectomy with removal of all mediastinal fatty tissue between the two bilateral phrenic nerves from the top of the horns of the thymus to the diaphragm.

### Treatment protocol for ph-PSL

In order to manage the risk of worsening MG symptoms due to steroid effects, oral PSL was initiated at 10 to 20 mg and gradually escalated to 100 mg on alternate days. Once patients had achieved stable disease status and had been maintained on high-dose PSL for two to four weeks, extended thymectomy was performed. When anti-cholinesterase was used as an initial treatment, it was reduced and discontinued after stabilization on PSL therapy. Histamine H2 blocker, vitamin D, and potassium were routinely administered to all patients for prophylaxis of adverse steroid effects. Postoperatively, the PSL dose was tapered by 5 mg per month over a minimum period of two years.

### Patient follow up and evaluation of outcomes

MG crisis was defined as an exacerbation of disease necessitating any of the following: (1) re-intubation, (2) >24-hour delay in extubation following surgery, or (3) additional plasma exchange. Data on other postoperative complications, including infection, wound dehiscence, diabetes mellitus, hyperlipidemia, osteoporosis, cataract, and depression, were also collected from the clinical records and evaluated.

All patients were followed for a minimum of three years. Postoperative MG status was assessed annually using the criteria cited by Jaretzki et al. [7]. “Improved” (Imp) was defined as a substantial decrease in pretreatment clinical manifestations or a sustained substantial reduction in MG medications. “Complete Stable Remission” (CSR) signified the absence of MG symptoms or signs for at least one year without treatment for MG during this period. Patients who met the former criteria for CSR but continued to take some form of treatment for MG were considered to have achieved “Pharmacologic Remission” (PR).

### Statistical analysis

The data were expressed as the mean and standard deviation. Differences between the treatment and control groups were examined using Fisher’s exact test and a chi-square test for categorical variables and the unpaired *t*-test for continuous variables. Survival analysis using the Kaplan Meier method was employed to examine the proportions of patients with Imp, CSR, and PR + CSR responses over time. The log-rank test was applied to detect the differences in the long-term outcomes between the groups. A *P*-value ≤ 0.05 was considered statistically significant.

### Ethics review

Prior to the study, the ethics review board of our institution examined and approved our research protocol as adhering to the Declaration of Helsinki.

## Results

### Patient characteristics

**Figure 1:**
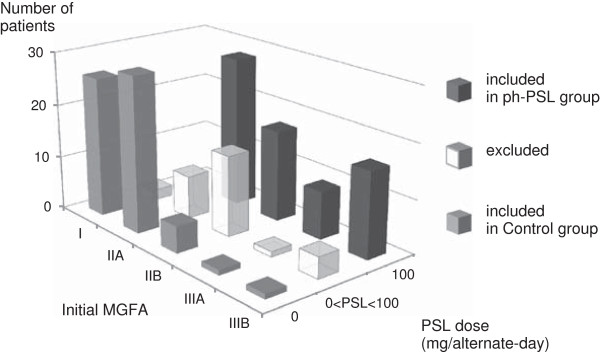
**Histogram of MG patients by prednisolone dose.** Values on the x-axis represent MGFA Class at the initial diagnosis of MG. Values on the y-axis indicate the dose of preoperative PSL administered on alternate days. The corresponding numbers of patients are represented by values on the z-axis. Black bars indicate patients who were included in the ph-PSL group. Gray bars signify those included in the control group, and white bars indicate patients excluded from the study due to low-dose prednisolone treatment.

Of the 180 patients with MG, 70 patients comprised the ph-PSL group. Sixty-one patients who did not receive any preoperative steroid treatment were included in the control group. The remaining 49 patients were excluded due to either treatment with PSL doses <100 mg on alternate days, previous record of treatments with steroids or immunosuppressive agents, MGFA Class ≥ IV, and/or insufficient information. Figure 1 shows the patients grouped by the inclusion criteria. Table 1 summarizes the baseline patient characteristics. At study entry, MG disease severity was significantly greater in the ph-PSL group compared to the control group (MGFA Class ≥ IIB, 42 [60.0%] versus 7 [11.3%], respectively; *P* < 0.001). Compared to controls, patients in the ph-PSL group demonstrated a significant reduction in the mean preoperative%FVC (92.4 ± 18.4% versus 99.5 ± 18.9%, respectively, *P* =0.037) as well as a marked increase in the mean preoperative FEV1.0% (85.2 ± 10.2% versus 81.4 ± 6.9%, respectively, *P* = 0.017). However, the two groups did not differ significantly with regards to other clinical features such as age, sex, serum level of acetylcholine receptor antibody, treatment with anti-cholinesterase and tacrolimus, the presence of thymoma, and operative procedure including thoracotomy with sternotomy or video-assisted thoracoscopic surgery.

**Table 1:** Patient characteristics

	**ph-PSL (n = 70)**	**Control (n = 61)**	***P*-value**
Age, years^†^	46.9 ± 17.4	51.3 ± 13.9	0.115
Sex, n, male/female	21/49	20/41	0.732
Preoperative%FVC^†^	92.4 ± 18.4	99.5 ± 18.9	0.037^*^
Preoperative FEV 1.0%^†^	85.2 ± 10.2	81.4 ± 6.9	0.017^*^
Initial MGFA Class I/IIA/IIB/IIIA/IIIB, n	0/28/17/9/16	25/29/5/1/1	
Initial MGFA Class I + IIA/IIB + IIIA + IIIB, n	28/42	54/7	<0.001^*^
Initial achR-Ab, nmol/l^†^	62.5 ± 98.4	93.0 ± 310.3	0.456
Preoperative anti-ChE, yes/no	47/23	33/28	0.127
Preoperative tacrolimus, yes/no	3/67	0/61	0.248
Thymoma, yes/no	21/49	27/34	0.091
Surgical approach, thoracotomy /VATS	54/16	52/9	0.272

^*^Statistically significant difference.

^†^Values represent mean ± standard deviation achR-Ab, acetylcholine receptor antibody; anti-ChE, anti-cholinesterase; FEV, forced expiratory volume; FVC, forced vital capacity; MGFA, Myasthenia Gravis Foundation of America; VATS, video-assisted thoracoscopic surgery.

### Clinical course of PSL treatments

**Figure 2:**
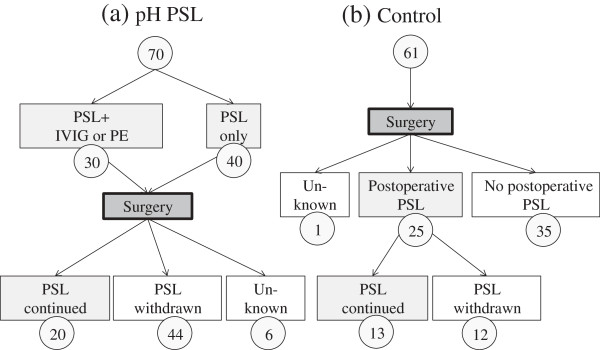
Clinical course of steroid treatments for study groups: (a) ph-PSL group; (b) control group.

Figure 2 shows the numbers of patients in each group receiving various types of treatment during the study period. In the ph-PSL group, 30 patients were treated with intravenous immunoglobulin and/or plasma exchange combined with PSL preoperatively, whereas 40 patients received ph-PSL only. Following surgery, PSL was withdrawn in 44 patients due to disease improvement while 20 patients continued on PSL. Although none of the patients in the control group received additional preoperative treatment with the exception of anti-cholinesterase, 25 patients in this group initiated PSL postoperatively due to worsening of MG.

### Preoperative stabilization of MG with PSL

**Figure 3:**
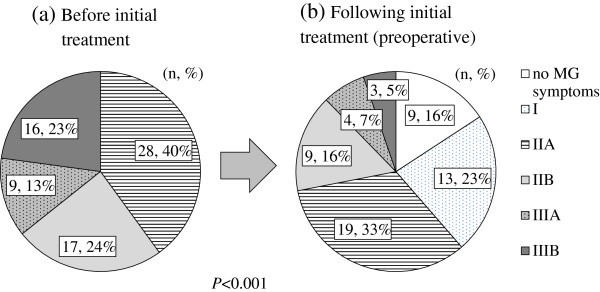
Distribution of the MG patients: (a) MGFA class at first presentation and (b) preoperative MGFA classes after the initial treatment.

In the ph-PSL group, MG status was well-stabilized during the preoperative period to the extent that in most cases, MGFA class was significantly improved (*P* < 0.001) (Figure 3) and anti-cholinesterase therapy was withdrawn. Of the 47 patients initiating anti-cholinesterase in combination with PSL, 18 patients discontinued anti-cholinesterase before surgery (cessation rate: 38.3%) and 24 discontinued treatment postoperatively (total cessation rate: 89.4%). In contrast, of the 33 controls commencing anti-cholinesterase therapy, only one patient (3%) was able to stop the medication preoperatively and 19 others discontinued treatment after surgery (total cessation rate: 60.6%). Overall, anticholinesterase therapy was withdrawn significantly more frequently in the ph-PSL group than in the control group during both the preoperative phase (*P* < 0.001) and the postoperative phase (*P* = 0.002).

### Postoperative complications including MG crisis

There were no intraoperative deaths in either group. Table 2 lists the frequency of postoperative complications occurring during hospitalization. Significantly fewer patients in the ph-PSL group experienced postoperative MG crisis compared to the control group (2 [2.9%] versus 8 [12.9%], respectively; *P* = 0.045). Pneumonia and wound infection accounted for three cases each in the control group but no such events occurred in the ph-PSL group (*P* = 0.098, *P* = 0.098, respectively). Four patients in the ph-PSL group and one patient in the control group developed wound dehiscence; however, the difference in the frequency between the groups was not significant (*P* = 0.368). Likewise, there were no significant differences between the groups in the frequency of steroid-related complications such as diabetes mellitus, hyperlipidemia, osteoporosis and cataract, following discharge (*P* = 0.303, *P* = 0.283, *P* = 1.000, *P* = 1.000, respectively) (Table 3).

**Table 2:** Postoperative surgical complications during hospitalization

	**ph-PSL (n = 70)**	**Control (n = 61)**	***P*-value**
Myasthenia gravis crisis	2	8	0.045^*^
Infection			
Pneumonia	0	3	0.098
Wound infection	0	3	0.098
Wound dehiscence	4	1	0.368

Values represent number of cases.

^*^Statistically significant difference.

**Table 3:** Postoperative steroid-related complications after discharge

	**ph-PSL**	**Control**	***P*-value**
	**(n = 70)**	**(n = 61)**	
Impaired glucose tolerance	3	6	0.303
Diabetes mellitus	3	6	0.303
Hyperlipidemia	6	2	0.283
Osteoporosis	2	1	1.000
Cataract	1	0	1.000

Values represent number of cases.

### Clinical outcomes

**Figure 4:**
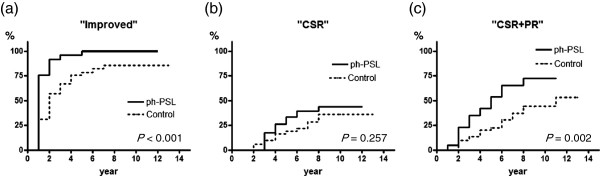
**Comparison of clinical outcomes (time to “Improved,” “CSR,” and “CSR + PR”) between study groups. (a)** “Improved,” **(b)** “CSR,” and **(c)** “CSR + PR”.

Patients were followed for a mean period of 6.9 ± 0.3 years. The rates of patients improved at three and five years were 92% and 96%, respectively, in the ph-PSL group compared to 57% and 76%, respectively, in the control group (*P* < 0.001) (Figure 4a). MG status among patients in the ph-PSL group improved more rapidly compared to the control group. The three-, five-, and ten-year rates of “CSR” were 0, 26%, and 44%, respectively, in the ph-PSL group compared to 6%, 16%, and 36%, respectively, in the control group (Figure 4b). Patients in the ph-PSL group tended to achieve CSR, but the proportions of patients attaining CSR at each interval did not differ significantly from those of the control group (*P* = 0.257). In contrast, significantly greater proportions of patients in the ph-PSL group achieved “CSR + PR” at three, five, and ten years (23%, 42%, and 72%, respectively) compared to controls (10%, 20%, and 44%, respectively; *P* = 0.002) (Figure 4c). Patients in the ph-PSL group appeared to achieve “CSR + PR” more readily than those in the control group.

## Discussion

Although patients receiving ph-PSL presented with greater disease severity compared to controls, the incidence of MG crisis was significantly lower in the former group due to effective preoperative stabilization of MG with high-dose PSL therapy. The frequency of steroid-related complications, including postoperative infection, wound dehiscence diabetes mellitus, hyperlipidemia and cataract, appeared to be similar in both groups. Furthermore, administering high-dose PSL therapy was more likely to improve postoperative MG status for a long-term period, suggesting that early induction with an immunosuppressant can modify the course of the disease.

MG crisis is the most severe phenotype, characterized by respiratory failure requiring invasive or noninvasive mechanical ventilation. Approximately 15 to 20% of patients with MG experience crisis in their lifetime, typically within the first two years of the diagnosis [8]. Alshekhlee et al. estimated that in a large US cohort, the overall in-hospital mortality rate in patients experiencing MG crisis was approximately 4.5%, which was higher than that in patients without MG crisis (2.2%). Along with other factors, including age and respiratory failure requiring endotracheal intubation, the diagnosis of MG crisis is a major predictor of mortality [9]. Because surgery is a recognized trigger, MG crisis is considered to be one of the most serious postoperative complications. Thus, preventing postoperative MG crisis is a key concern among general thoracic surgeons and neurologists.

Accordingly, the effectiveness of preoperative steroidal treatment for MG patients has been investigated for several decades [10,11]. The rationale for implementing induction steroid therapy combined with extended thymectomy may be explained as follows. An induction of steroidal agents attenuates serum AchR-Ab and stabilizes MG status [12]. With stabilization, anti-cholinesterase treatment can be withdrawn or reduced. In turn, discontinuation of anti-cholinesterase revives and stabilizes the function of AchR. Stabilizing MG status allows invasive surgical treatment to be performed safely.

Johns et al. reported favorable outcomes for MG patients who were treated with preoperative prednisone in combination with thymectomy [13]. Several other studies have evaluated various steroid regimens as well. Warmots et al. and other researchers reported high response rates and lower morbidity for patients treated with an alternate-day regimen of prednisone [14]. Seybold et al. purported that gradually increasing the dose of prednisone during therapy reduced the risk of worsening disease in MG patients initiating steroid treatment [3]. Combining these approaches, Bolooki et al. found that high-dose steroid therapy was efficacious in the perioperative management of MG patients undergoing thymectomy [15]. Recently, Klimek-Piotrowska et al. reported the findings of a prospective autopsy study and confirmed that ectopic thymic tissues frequently exist in the mediastinum beyond the confines of the surgical field [16], suggesting a limitation of surgical approaches for this disease and a necessity for multimodal therapy.

Based on the findings of these previous studies, we started offering perioperative high-dose PSL therapy followed by extended thymectomy in 1978 for MG patients in order to stabilize perioperative MG status, prevent postoperative MG crisis, and achieve early disease remission. Our experience, as presented in this study, provides further evidence supporting the effectiveness of this therapy.

It is important to examine the frequency of steroid side effects in our study. Although we found these types of complications in the ph-PSL group, the incidence of such adverse effects was nearly identical to that in the control group (Tables 2 and 3). Numerous studies examining the outcomes of perioperative steroid therapy during thymectomy for MG patients have reported similar results [4,6,17,18]. We offer several possible explanations for these findings. Complications during hospitalization such as wound infection or wound dehiscence may have been attributed to the comparably lower cumulative dose of PSL. The induction of the PSL treatment commenced about two months prior to surgery, and the total cumulative dose of PSL at the time of surgery was approximately 2,000 mg. According to a study of ulcerative colitis patients, total prednisolone doses ≥10,000 mg may be a risk factor of surgical site infection [19]. Compared to colorectal surgery, thymectomy is less likely to be associated with infection due to the different environment of microorganisms. Thus, at the time of surgery, the cumulative dose of PSL may not have been excessive for this patient group. Regarding complications following discharge, such as steroid-induced diabetes, it has been previously reported that total glucocorticoid dose and duration of therapy are strong predictors of diabetes induction [20]. However, a study of patients with neurologic disease by Iwamoto et al. found no difference in the cumulative PSL doses between patients with or without steroid-induced diabetes [21]. The authors suggested that when patients received high-dose, short-term steroid therapy, cumulative doses of steroids were minimally associated with adverse effects. However, a precise mechanism was not described. Nevertheless, appropriate management of patients receiving steroid therapy is important. We deem that administering a Histamine H2 blocker and vitamin D, conducting routine tests for glucose tolerance, and offering medical advice might assist in preventing steroid-induced diabetes. In this study, steroid-related complications did not occur.

On the other hand, some clinicians advocate initiating steroid therapy only after an exacerbation of MG, due to concerns over steroid side effects [22]. We disagree with this point of view because delayed immunosuppression may produce a less complete response [23,24]. Indeed, in the control group of our study, 25 of 61 (40%) patients received steroids after thymectomy due to unstable postoperative disease, and half had continued the medication at the time of our investigation. Once MG exacerbation occurs, patients are likely to be resistant to treatments. Our study demonstrated that contrary to these opinions, perioperative high-dose PSL therapy was effective and led to fewer steroidal side effects. We strongly believe that preoperative induction with high-dose PSL therapy should be recommended as part of the surgical protocol.

However, routine administration of high-dose PSL followed by thymectomy for all MG patients without any consideration is not justified. For example, during the early phase of the disease in mild MG cases, the germinal centers in thymus are not developed, and therefore, preoperative steroid therapy might not be required [25]. Moreover, even thymectomy for class I MG remains controversial [26]. In fact, most cases in the control group did not experience crisis or exacerbation of MG even when they had not received any preoperative PSL therapy. In our institution, the criteria for instituting PSL therapy in surgical MG patients are based on patient characteristics, disease severity, and the presence of advanced thymoma. Decisions regarding induction therapy should be made only after careful assessment of these factors.

The appropriateness of using an alternate-day regimen of PSL 100 mg for immunosuppression must also be determined. This regimen was implemented based on information in the literature and our experience but it has not been validated by clinical trials. Although the frequency of steroid-related adverse events in the ph-PSL group in this study was similar to that in the control group, the efficacy and minimum dose required for PSL should be further investigated. Currently, we are conducting an ongoing clinical study in order to evaluate whether an induction of tacrolimus can reduce the dosage of PSL in MG surgical patients.

Authors from our group have previously reported the outcomes of PSL therapy [4,6]; likewise, researchers from other institutions have produced similar reports [17,18]. Our present findings strengthened the evidence supporting the clinical benefits of administering high-dose PSL perioperatively in conjunction with extended thymectomy. Comparing a large sample of MG patients with or without preoperative PSL therapy is also advantageous over extant published studies, wherein comparisons were not performed, or if so, the studies employed inconsistent dosages of PSL and smaller sample sizes.

This study is limited by its retrospective design. Although we employed specific eligibility criteria for the high-dose PSL therapy, some amount of selection bias existed. However, our research may be justified for two reasons. First, in this comparative study, the ph-PSL group, which represented a disadvantaged patient population with more severe disease, demonstrated superior outcomes with regard to the development of MG crisis. Thus, despite the differences between the patient groups, the results of this study are nevertheless noteworthy. Second, long-term follow-up of MG patients requires a protracted period of time, making a longitudinal study difficult to conduct. However, plans for a prospective study in currently underway, as described previously. Nevertheless, we believe that this present study offers supportive evidence and noteworthy information.

In clinical practice, the dosage of PSL varied depending on the patients’ characteristics, MG status, and concomitant diseases (Figure 1). Although most cases receiving steroid therapy were treated with an alternate-day regimen of 100 mg, some had received 90 mg or less. To avoid ambiguity in the data, the inclusion criteria for the ph-PSL group specified a PSL dose of 100 mg on alternate days, whereas patients in the control group who received lower PSL doses and those who had received previous steroidal therapy were excluded.

## Conclusion

Perioperative high-dose PSL therapy is a promising strategy for managing MG patients in terms of reducing the incidence of postoperative MG crisis and providing long-term disease control. However, a prospective randomized study in a multicenter setting is warranted in order to confirm the efficacy and safety of this approach.

## Abbreviations

MG: Myasthenia gravis; AchR-Ab: Acetylcholine receptor antibodies; AchR: Acetylcholine receptor; PSL: Prednisolone; MGFA: Myasthenia Gravis Foundation of America; ph-PSL: Perioperative high-dose PSL; VATS: Video assisted thoracoscopic surgery; Imp: Improved; CSR: Complete stable remission; PR: Pharmacologic remission.

## Competing interests

The authors declare that they have no competing interests.

## Authors’ contributions

YY participated in research design and data analysis, and drafted the manuscript; SY participated in research design and helped to draft the manuscript; HS, TT, TI and TM participated in the clinical practice and the data analysis; NK participated in the clinical practice, its design, and coordination; IY conceived the study, participated in its design and coordination, and helped to draft the manuscript. All authors read and approved the final manuscript.
